# Evaluating the performance of Fourier transform infrared spectroscopy for typing and outbreak investigation of methicillin-resistant and -susceptible *Staphylococcus aureus*

**DOI:** 10.1128/spectrum.03245-25

**Published:** 2026-04-14

**Authors:** Jaakko Silvola, Inka Harju, Teemu Kallonen, Kirsi Gröndahl-Yli-Hannuksela, Mari Kanerva, Jaana Vuopio, Kaisu Rantakokko-Jalava

**Affiliations:** 1 Clinical Microbiology Laboratory, Turku University Hospital 1041, Turku, Finland; 2 Institute of Biomedicine, University of Turku 169300, Turku, Finland; 3 Infection Control Unit, Turku University Hospital 1041, Turku, Finland; 4 Microbiology Unit, Finnish Institute for Health and Welfare 3837, Helsinki, Finland; LSU Health Shreveport, Shreveport, Louisiana, USA

**Keywords:** healthcare-associated outbreak, MRSA, *S. aureus*, IR Biotyper

## Abstract

Infections caused by methicillin-susceptible *Staphylococcus aureus* (MSSA) and methicillin-resistant *Staphylococcus aureus* (MRSA) can be life-threatening and complicated to manage. Especially in the healthcare environment, MRSA is a notorious cause of outbreaks inflicting a significant disease burden. Assessing the relatedness of isolates is a key challenge for outbreak management. We aimed to assess the utility and performance of Fourier transform infrared (FTIR) spectroscopy (IR Biotyper; Bruker Daltonics GmbH & Co. KG, Bremen, Germany) by comparing it with *spa* typing and whole-genome sequencing (WGS). *S. aureus* was cultured under standard conditions on Müller-Hinton agar, and the second subculture was used for spectral acquisition. Eighty-eight MRSA isolates and 89 MSSA isolates were included from both screening and clinical MRSA specimens, and blood culture MSSA specimens were identified at the clinical microbiology laboratory of Turku University Hospital, Southwest Finland. Among non-outbreak strains, two or three possible subtypes were detected with FTIR within 16 different *spa* types. Two suspected healthcare-associated outbreaks comprising nine and four isolates, respectively, were detected. WGS confirmed the outbreak with four isolates. Within the other outbreak, WGS revealed five closely related and four unrelated isolates. FTIR analysis correctly differentiated two of the unrelated isolates, while two unrelated isolates were erroneously clustered in a principal component analysis. Although the discriminatory power of FTIR was lower than WGS, false-negative clustering was not observed. FTIR showed a lower analysis cost per specimen and was superior in processing speed to both WGS and *spa* typing. Our findings suggest the potential use of FTIR in preliminary screening, supporting almost real-time outbreak analysis and hence outbreak management.

**IMPORTANCE:**

In this study, we evaluated the performance of Fourier transform infrared (FTIR) spectroscopy for the typing of both methicillin-resistant *Staphylococcus aureus* (MRSA) and methicillin-susceptible *Staphylococcus aureus* (MSSA) isolates in comparison with *spa* typing, as well as outbreak analysis of MRSA isolates in comparison with *spa* typing and whole-genome sequencing (WGS). FTIR spectroscopy can be used to detect phenotypic variation of *S. aureus* isolates sharing the same *spa* type, which could be used as screening to detect possible new circulating MRSA and MSSA strains. We were able to infer clinically meaningful information on the relatedness of MRSA isolates for outbreak management with FTIR, which showed good concordance to WGS-based typing. Although 2 out of 13 MRSA isolates were erroneously clustered in a principal component analysis, no false-negative clustering was observed. FTIR spectroscopy continues to show promise as an emerging and cost-effective strain typing tool for both MRSA and MSSA isolates.

## INTRODUCTION

*Staphylococcus aureus* is a common colonizer of humans and the environment and a significant opportunistic pathogen responsible for infections of variable severity (1). Methicillin-susceptible *Staphylococcus aureus* (MSSA) and methicillin-resistant *Staphylococcus aureus* (MRSA) strains remain a persistent challenge attributing major burden of disease both in healthcare facilities and the community (2, 3). Laboratory-based bacterial typing is crucial for MRSA outbreak detection and investigations, as well as for epidemiological studies to assess and limit the impact of multi-drug resistant microorganisms on healthcare systems and the community (4). While the existing tools have been developed and experimentally validated for the prevention of MRSA, the lack of feasible typing strategies for MSSA isolates currently limits the understanding of the relationship between MRSA and MSSA isolates. Bacterial strain typing is often performed in expert laboratories where the cost and time of reporting can vary significantly. A need for accessible, quick, and cost-effective typing methods has been recognized.

In Finland, *spa* typing is performed on all new MRSA cases by the Finnish Institute for Health and Welfare (THL), complemented by outbreak-oriented, targeted whole-genome sequencing (WGS) (5). WGS has emerged as the gold standard method for high-resolution *S. aureus* strain typing, producing reliable and reproducible phylogenetic relationships (6). WGS has enabled detection of variants within a *spa* type that differ with up to 3,500 single-nucleotide polymorphisms (SNPs) (7). *spa* typing is usually done with Sanger sequencing because of cost and the challenges tandem repeats can provide when assembling short reads (8). Recently, the IR Biotyper (Bruker Daltonics GmbH & Co. KG, Bremen, Germany) has been proposed as both a stand-alone typing tool and a high-throughput screening tool for isolates selected for WGS, thereby possibly reducing the time and cost associated with extensive sequencing (9).

The IR Biotyper utilizes Fourier transform infrared (FTIR) spectroscopy technology to discriminate bacterial strains (10). The FTIR method is based on the absorption of infrared light by different biomolecules present in the sample, followed by the production and comparison of the absorption spectra, where peaks in certain wavenumber regions (spectral windows) can be correlated with known structures (e.g., cell-wall carbohydrates) (11). After spectral acquisition and bioinformatic preprocessing, a spectral window can be chosen for differentiating both gram-negative and -positive bacteria on the subspecies level, using either hierarchical clustering or multi-dimensional plotting strategies (12). Another approach adopted in the IR Biotyper is generating classifiers, which can be used to predict phenotypic typing results, such as serotypes, of unknown isolates (13, 14). In this study, only hierarchical clustering and multi-dimensional plotting (principal component analysis [PCA]) strategies are used to differentiate *S. aureus* strains.

FTIR has been used as a typing method for outbreak detection of *S. aureus*, and sufficient discriminatory power in comparison with WGS has been observed in some studies (9, 15, 16). In a data set of 70 strains, FTIR was able to achieve discriminatory power comparable to *spa* typing and pulsed-field gel electrophoresis (17).

In this study, we aimed to evaluate the performance of the IR Biotyper for *S. aureus* strain typing in the clinical microbiology laboratory of Turku University Hospital (TYKS) in Southwest Finland. We assessed whether the FTIR method could be used to identify *S. aureus* subtypes by combining it with *spa* typing and evaluated its performance in outbreak analysis in comparison with WGS.

## MATERIALS AND METHODS

### Bacterial isolates

*S. aureus* isolates in this study were collected prospectively in the clinical microbiology laboratory at the TYKS, a tertiary care hospital in Southwest Finland with a catchment population of 494,819 by the end of 2024 (18). Isolates were stored in the strain bank of the clinical microbiology laboratory in TYKS. The collection included one isolate/patient: (i) all new *S. aureus* isolates from diagnostic blood cultures and (ii) all new MRSA isolates from all diagnostic clinical (blood, superficial/deep skin swab, and urine) and screening specimens during a 6-month period (September 2023–February 2024). MSSA isolates were sent for *spa* typing to the Institute of Biomedicine, University of Turku. MRSA isolates were sent to THL for *spa* typing.

Information on the sampling date, specimen type, and association with a suspected outbreak was collected for each isolate. The data set was pseudonymized. All new MRSA isolates were reported via phone to the infection control unit of Turku University Hospital, where suspected outbreaks were actively identified and managed. For this study, the infection control unit reported suspected healthcare-associated MRSA outbreaks to the investigators via phone or secure e-mail. Suspected MRSA outbreaks were defined as two or more cases identified in the same healthcare unit, ward, or long-term care facility during the isolate collection period, and at least one of the cases had to be sampled during the collection period.

Positive blood cultures were detected with the BACTEC FX (BD, Franklin Lakes, NJ, USA) system, and *S. aureus* was identified with standard methods, including a rapid coagulase test (Staphaureus Plus; Remel, San Diego, CA, USA) and MALDI-TOF MS (Bruker Daltonics GmbH & Co. KG). MRSA was identified with either cefoxitin disk in antimicrobial susceptibility testing according to EUCAST standard or directly from clinical specimens with the eazyplex MRSA Kit (Amplex Diagnostics GmbH, Gars, Germany). From screening samples, MRSAs were detected using an enrichment broth (eMRSA; Copan Diagnostics, Murrieta, CA, USA) and a selective chromogenic agar (Chromagar MRSA II, BD).

### Culture conditions

*S. aureus* strains were stored in broth with 15% glycerol at −80°C. From the primary diagnostic culture or from storage tubes, the strains were inoculated with a 1 μL plastic loop to Müller-Hinton agar (MH) plates for FTIR and tryptic soy agar supplemented with 5% sheep blood for DNA extraction and incubated under standard conditions (18 hours in 35°C, 5% CO_2_). For FTIR, the isolates were subcultured on MH plates on the next day, and the standard incubation (18 hours in 35°C, 5% CO_2_) was repeated. The subculture was used for spectral acquisition.

### *spa* typing and WGS

DNA was isolated using a NucleoSpin Microbial DNA Kit (Macherey-Nagel, Düren, Germany). All isolates were *spa* typed as described previously (19). *spa* types were assigned using web-based tools spaTyper (https://spatyper.fortinbras.us/) and Ridom *spa* server (https://spa.ridom.de/). Sequencing libraries were prepared using the Nextera XT Library Prep Kit (Illumina, San Diego, CA, USA). WGS was performed on the outbreak isolates on the NextSeq 2000 System (Illumina) platform using 300 bp paired-end reads. Sequences were assembled *de novo* and mapped to a reference genome (GenBank: CP014791.1) using the CLC Microbial Genomics Module (v24.1.1; Qiagen Digital Insights, Aarhus, Denmark). A maximum-likelihood phylogeny and a single-nucleotide variant (SNV) matrix were inferred using standard parameters (ignoring MNVs, Jukes Cantor substitution model, substitution rate variation) on the SNP Tree tool (20). Sequence types were called from the sequencing reads using a k-mer-based tool integrated into the CLC software. A conservative clustering threshold to include transmission events within 6 months was 15 SNVs, while isolates within 16–49 SNVs were defined as related and >50 SNVs as unrelated (21, 22). *spa* typing of MRSA isolates was performed by THL, while WGS of outbreak isolates and *spa* typing of MSSA isolates were conducted in the Institute of Biomedicine, University of Turku. iTOL software was used for data visualization (23).

### Spectral acquisition

Preparation of the bacterial isolates for spectral acquisition with the IR Biotyper was conducted according to the manufacturer’s instructions, with a slight modification in reagent volume to ease the suspension homogenization. Briefly, a 1 μL plastic loop was used to carefully collect biomass from the culture plate. Biomass was suspended into 100 μL of 70% (vol/vol) ethanol, and the suspension was vortexed rigorously until it was visually homogenous. One hundred microliters of LC/MS grade water was added, and the suspension was again briefly vortexed. Fifteen microliters of suspension was pipetted to sample spots of a 96-well silicon microtiter plate, where analysis of each spot results in one IR spectrum. To ensure technical reproducibility, each sample was pipetted to four different spots (technical replicates), of which we aimed to acquire three spectra passing the quality control (QC) parameters. The plate was dried for 10–15 minutes at room temperature before spectral acquisition. Two *Escherichia coli* reference strains (IRTS 1 and IRTS 2, Bruker) were used as a control in each run. Before placing the plate in the IR Biotyper instrument, the plate was visually inspected for signs of under- or overdried sample spots. Each isolate was analyzed on at least two separate days from independent culture cycles (biological replicates), resulting in six spectra per isolate for downstream analysis. The ATCC 25923 strain was used to validate between runs.

### Spectral analysis

Sample spots failing to meet the preprogrammed QC parameters on the instrument were ignored, and the acquisition step was repeated, if necessary. Six spectra passing the quality control, including three technical and two biological replicates of each strain, were randomly selected and included in the downstream analysis. Preprocessing of the spectra was done automatically after spectral acquisition with the Bruker IR Biotyper (v4) and the underlying OPUS software. The default acquisition range between wavenumbers 1,300 and 800 cm^−1^ was used to calculate distance values with the Euclidean method. Hierarchical cluster analysis (HCA) with the ward’s algorithm was used in the analysis of the outbreak strains, and PCA model with a target to include 95% of the variance in the data set was used for exploratory plotting. Analysis and visualization of spectra were performed with Quasar (v.1.11) (24). Clustering cutoff in HCA was assessed by optimizing the coherence of each isolate and visually inspecting that each isolate was separated. For a supervised analysis of the outbreak isolates, the PAST (v.5.2) software was used to conduct a linear discriminant analysis (LDA) (25). Using the extracted principal components, we performed LDA using the isolate name as grouping identifier. The LDA centroid axes (mean value of the six LDA axes per isolate) were used in HCA (Ward’s linkage algorithm) to infer a dendrogram of the outbreak isolates.

## RESULTS

### Isolate collection

A total of 178 staphylococcal isolates were collected between September 2023 and February 2024. One isolate (SWF_90) turned out to be *Staphylococcus argenteus* in WGS and was discarded from the data set (total number of *S. aureus* isolates being thus 177). Of these, 88 were MRSA and 89 MSSA. As per inclusion criteria, all MSSA isolates were derived from clinical blood cultures, while MRSA isolates were derived from 54 screening specimens, 28 wound swabs, 4 blood cultures, and 2 urine cultures.

### *spa* typing

All 177 *S. aureus* isolates were *spa* typed, resulting in 86 different types overall. MRSA isolates were distributed to 41, and MSSA isolates to 58 different *spa* types. Thirteen *spa* types were identified from both MRSA and MSSA isolates.

The most frequent *spa* types overall were t359 (10 isolates), t127 (10 isolates), and t008 (nine isolates), all predominantly MRSA isolates (Table 1). The most frequent *spa* type associated with MSSA isolates was t267 (five isolates). Among 18 *spa* types identified in two to three isolates, as well as among the 56 singletons, MSSA isolates were more abundant. The greater diversity of MSSA isolates in this data set reflects the different inclusion criteria of MRSA and MSSA isolates: MRSA isolates included specimens from active inpatient screening as well as sporadic clinical specimens, whereas MSSA isolates were collected from unselected cases of *S. aureus* bacteremia.

**TABLE 1:** *spa* type distribution between MRSA and MSSA isolates, number of possible subtypes identified with FTIR within isolates of the same *spa* type, differentiation of MRSA and MSSA, or specimen types between the possible subtypes and corresponding supplementary figure of the detailed analysis

*spa* type(s)	Total, *n* (%)^*a*^	MRSA isolates, *n* (%)^*b*^	MSSA isolates, *n* (%)^*b*^	Possible subtypes (*n* isolates per subtype)	Differentiation by methicillin susceptibility between subtypes	Differentiation by specimen type between subtypes	Supplementary figures
t359	10 (5.6)	9 (90)	1 (10)	2 (9, 1)	Yes	No	S1
t127	10 (5.6)	7 (70)	3 (30)	2 (2, 8)	No	No	S2
t008	9 (5.1)	6 (66.7)	3 (33.3)	3 (6, 2, 1)	No	No	S3
t304	7 (4.0)	7 (100)	0 (0)	2 (5, 1)^*d*^	Only MRSA	No	S4
t386	7 (4.0)	7 (100)	0 (0)	2 (1, 6)	Only MRSA	No	S5
t172	7 (4.0)	3 (42.9)	4 (57.1)	2 (6, 1)	No	No	S6
t692	6 (3.4)	3 (50)	3 (50)	3 (1, 2, 3)	Yes	No	S7
t267	6 (3.4)	1 (16.7)	5 (83.3)	2 (4, 1)^*d*^	No	No	S8
t002	5 (2.8)	1 (20)	4 (80)	3 (1, 2, 2)	No	No	S9
t355	4 (2.3)	4 (100)	0 (0)	1	Only MRSA	No	S10
t015	4 (2.3)	0 (0)	4 (100)	3 (1, 1, 1)^*d*^	Only MSSA	Only BC	S11
t084	4 (2.3)	0 (0)	4 (100)	3 (1, 1, 2)	Only MSSA	Only BC	S12
6 *spa* types with 3 associated isolates each^*c*^	18 (10.2)	10 (55.6)	8 (44.4)	t021: 2 (1, 1)^*d*^ t437: 2 (1, 1)^*d*^ Others: 1	t021: yes t437: only MRSA Others: only MRSA/MSSA	t021: no t437: no Others: no	S13
12 *spa* types with 2 associated isolates each^*c*^	24 (13.6)	10 (41.7)	14 (58.3)	t362: 2 (1,1) t1309: 2 (1,1) Others: 1	t362: only MSSA t1309: yes Others: only MRSA/MSSA	t362: only BC t1309: yes Others: no/only BC	S14
Singletons^*c*^	56 (33.3)	21 (37.5)	36 (64.3)	3 (2, 2, 1)	–^*e*^	–	S15

^*a*^ % of all isolates.

^*b*^ % within *spa* type.

^*c*^ See Table S1 for detailed distribution.

^*d*^ Non-typeable isolate due to high variation between biological/technical replicates.

^*e*^ –, not applicable.

### FTIR

**Fig 1:**
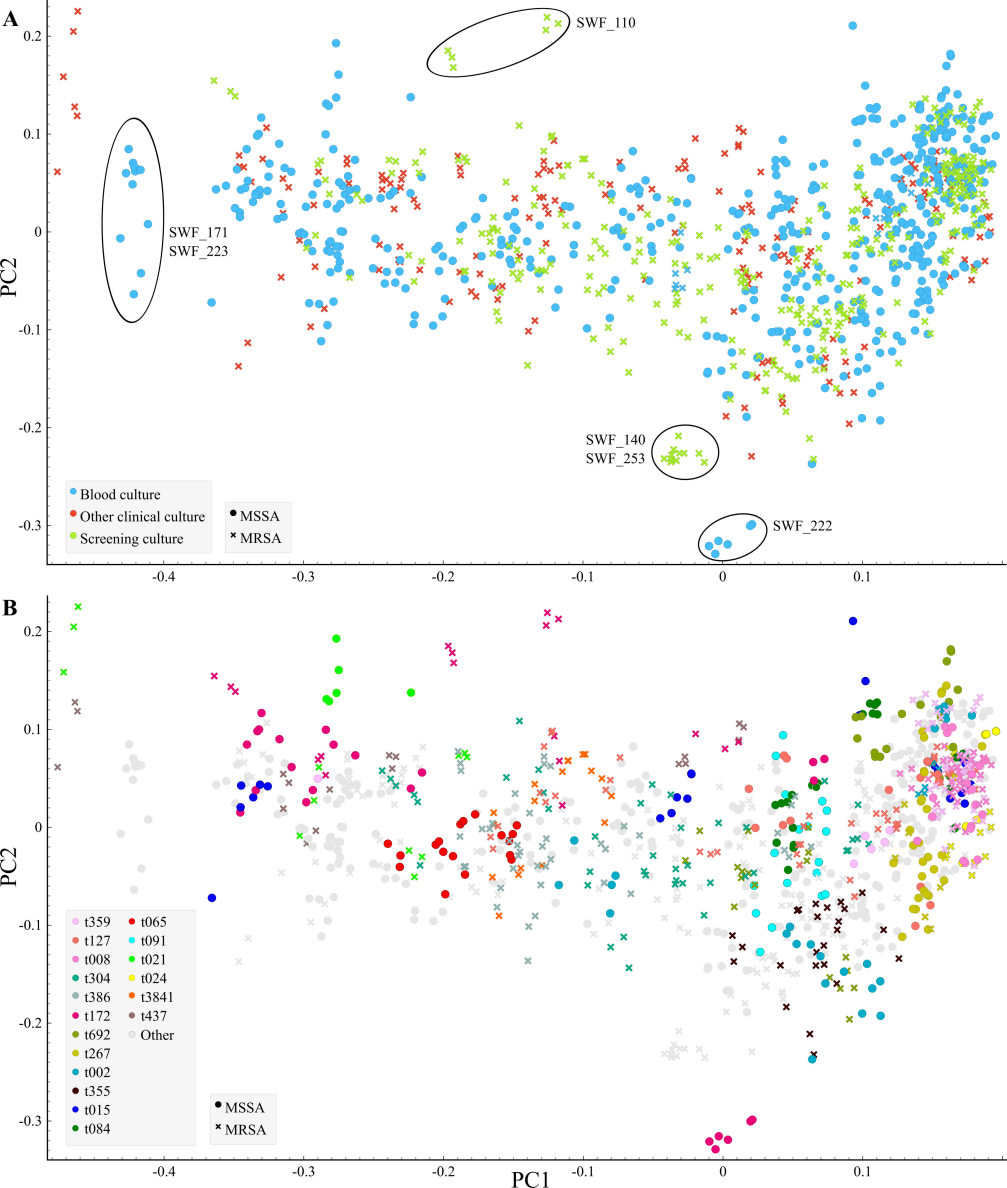
Two-dimensional scatter plot of the principal component analysis model of *S. aureus* isolates (*n* = 176) annotated by (**A**) specimen type (color) and methicillin susceptibility (shape) or (**B**) *spa* type (color, types with two isolates or less in gray: other). Each dot represents an absorption spectrum, six replicate spectra per isolate. Wave number region 1,300–800 cm^−1^. Phenotypic outlier isolates (SWF_110 and SWF_222) and subclusters (SWF_140, SWF_253 and SWF_171, SWF_223) outlined.

All 177 *S. aureus* isolates were analyzed with FTIR. One isolate was removed due to very high technical variability (SWF_245). Six spectra from each isolate, acquired with the FTIR, were included (1,056 individual spectra) and analyzed with PCA (Fig. 1). In the PCA model including all isolates, MRSA and MSSA isolates shared a high degree of phenotypical similarity. Only two outlier isolates could be delineated, namely, a blood culture MSSA isolate (SWF_222) and a screening MRSA isolate (SWF_110). Possible clusters of two isolates each could also be outlined, including blood culture MSSA isolates SWF_171 and SWF_223 and screening MRSA isolates SWF_140 and SWF_253.

To identify possible subtypes, the PCA model was applied to isolates sharing the same *spa* types (Fig. S1 to S14). Possible subtypes were identified with this approach among 16 *spa* types, while six isolates were considered non-typeable due to high variation between biological or technical replicate spectra (Table 1). MRSA and MSSA isolates among four different *spa* types were separated between possible subtypes, whereas an overlap of spectra from both MRSA and MSSA isolates was observed across five different *spa* types. Among the four *spa* types where MRSA and MSSA isolates were separated, the isolates did not differ by specimen types, although MRSA isolates mostly originated from non-invasive specimens and MSSA isolates from invasive specimens.

### Outbreak analysis

Two suspected MRSA outbreaks were epidemiologically identified, comprising nine (suspected outbreak A [SOA]) and four (suspected outbreak B [SOB]) isolates, respectively. The suspected outbreaks originated from the same healthcare facility, SOA followed by SOB, with approximately 6 months between the detection of index cases. WGS and *spa* typing were used to determine the relatedness of the suspected outbreak isolates. To evaluate the performance of the IR Biotyper, absorption spectra of isolates from the suspected outbreaks were clustered by both PCA and HCA models, and the partitions were compared to analysis with *spa* typing and WGS.

**Fig 2:**
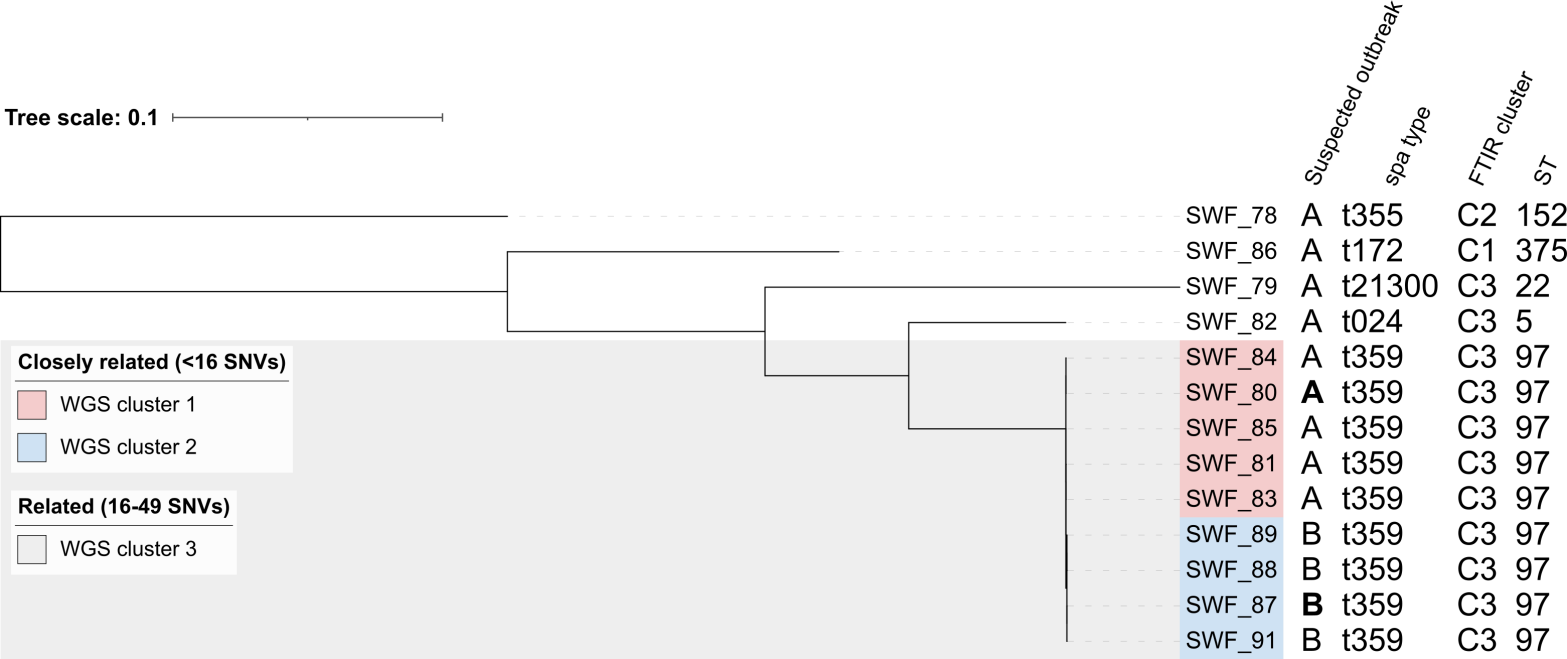
SNV-based dendrogram of the suspected outbreak isolates (*n* = 13), annotated by suspected outbreak association (A or B), *spa* type, FTIR cluster, and sequence type (ST). Related isolates (16–49 SNVs) and closely related (<16 SNVs) isolate clades are highlighted. Index cases are shown in bold.

Among SOA isolates, five isolates exhibited *spa* type t359, and four isolates exhibited different *spa* types each. All SOB isolates exhibited t359 (Fig. 2). WGS confirmed the close relatedness of the five t359 isolates within SOA (median pairwise distance 9 SNVs, range 4–13 SNVs; Table S2), while the four other isolates in SOA were considered unrelated (pairwise distance >49 SNVs). Within SOB, all isolates were considered closely related (median pairwise distance 10 SNVs, range 8–13 SNVs). Additionally, between the t359 isolates of SOA and SOB, WGS analysis revealed a moderate degree of relatedness (median pairwise distance 39 SNVs, range 31–47 SNVs), suggesting a recent link between the suspected outbreaks, which is supported by the origin of the outbreaks in the same healthcare facility.

**Fig 3:**
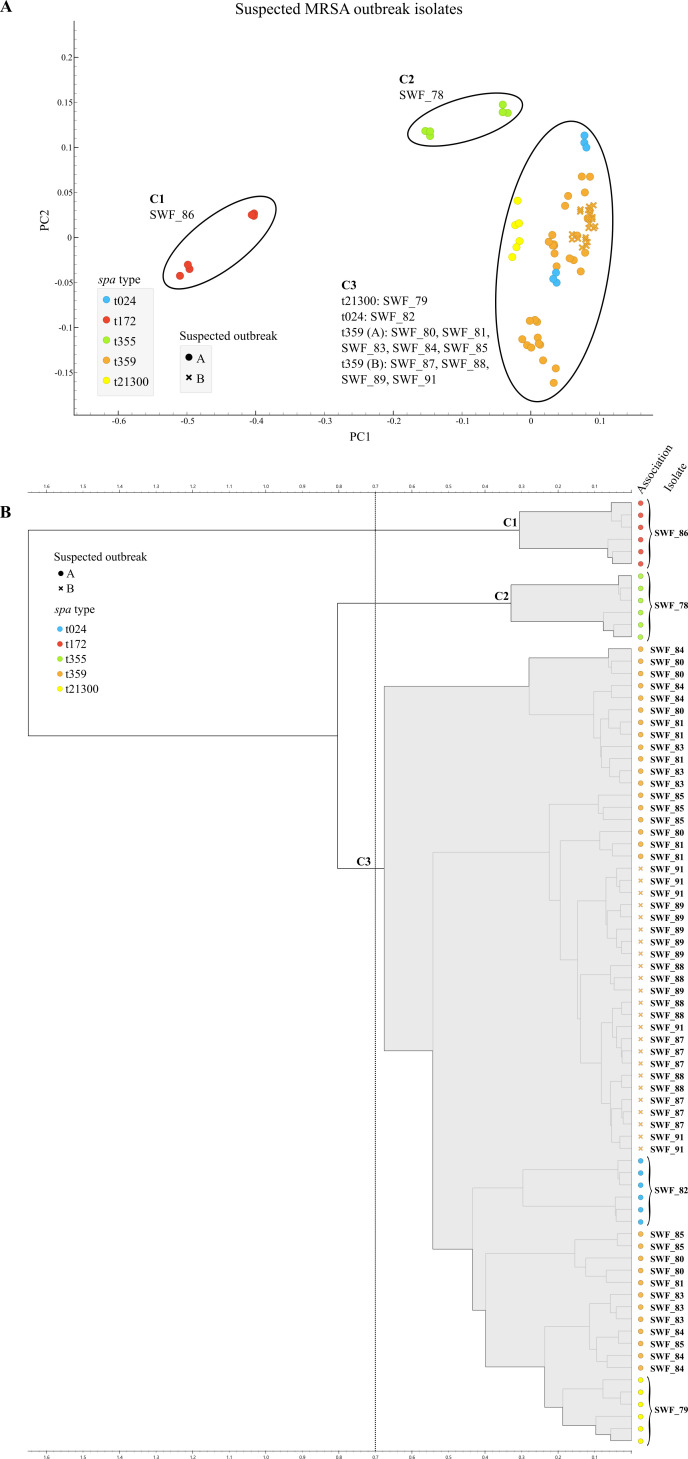
(**A**) Suspected MRSA outbreak isolates. Two-dimensional scatter plot of the PCA model annotated by *spa* type and suspected outbreak association (*n* = 13), six spectra per isolate. Each dot represents an absorption spectrum in the wave number region 1,300–800 cm^−1^. Three possible clusters outlined (C1–C3), including outlier isolates SWF_86 (C1) and SWF_78 (C2), possibly separated from the cluster of other suspected outbreak isolates (C3). Overlap of spectra from both suspected outbreaks observed in C3. (**B**) Hierarchical cluster analysis of the HCA model annotated by *spa* type and suspected outbreak association (*n* = 13), six spectra per isolate. Euclidean distances and the Ward’s linkage algorithm. Clustering (C1–C3) cutoff was set to maximize cluster coherence (i.e., including all spectra of a single isolate). Suspected outbreak B forming a subcluster within C3 otherwise comprising isolates of suspected outbreak A.

In the PCA, two phenotypic outlier isolates (t172-SWF_86 and t355-SWF_78) were separated from the cluster of highly similar isolates, including isolates from both suspected outbreaks (Fig. 3A). The same result was obtained with the HCA approach (Fig. 3B). However, two unrelated isolates of SOA (t21300-SWF_79 and t024-SWF_82) were clustered with the other outbreak isolates, indicating two false-positive typing results with the FTIR PCA method in comparison to both sequence-based methods. However, using a supervised LDA analysis where each isolate was averaged to one centroid axis to reduce technical variation, the unrelated isolates (t21300-SWF_79 and t024-SWF_82) were separated from the other suspected outbreak isolates (Fig. S16).

## DISCUSSION

This study aimed to evaluate the utility and performance of the FTIR methodology as a tool for detecting related *S. aureus* strains, with a focus on utility in outbreak investigation. We applied FTIR to 177 *S. aureus* isolates and detected an overall high degree of phenotypic similarity between MRSA and MSSA isolates collected from Southwest Finland during a 6-month period. A substantial overlap of isolates with different *spa* types was observed in the PCA (Fig. 1). This indicates that, with this approach, FTIR may not be suitable for routine surveillance but is limited to the analysis of suspected outbreaks and variation within *spa* type. The high degree of similarity is unsurprising due to the different determinants of methicillin susceptibility (target mutation of a cell-wall transpeptidase) and a typing result in FTIR analysis (spectral window corresponding to surface glycopolymer region) (12).

Because the FTIR typing result originates from phenotypic differences, our findings suggest the presence of two or multiple phenotypic subtypes among 16 circulating *S. aureus* strains sharing the same *spa* type. This is likely because the genetic content of *S. aureus* strains exhibiting similar *spa* types can also be variable (26). For example, given the results of our outbreak analysis and the previously reported adequate negative predictive value of FTIR, the t359 MSSA isolate in our data set was very likely not related to the MRSA outbreak (9). Regarding other *spa* types, the outlier subtypes in our FTIR analysis were also frequently blood culture MSSA isolates, which probably harbor different determinants of virulence from colonizing MRSA strains (Fig. S1 to S4). Differences in the ability of FTIR to type *S. aureus* isolates may also be lineage specific or may arise from analysis strategies, as no gold standard for this methodology in clinical microbiology is currently established (17, 27). The limitation of this study was the lack of WGS confirmation of the subtypes detected with FTIR for most of the isolates.

In the outbreak analysis, FTIR with PCA resulted in false-positive clustering of two unrelated isolates (Fig. 2 and 3). This result indicates that non-invasive MRSA isolates can exhibit a similar surface phenotype, although they are genetically unrelated, as is suggested also by the FTIR clustering by *spa* type. Another explanation can be technical variation, as in the LDA, a similar partition to WGS was observed (Fig. S16). For outbreak analysis of MRSA isolates, molecular methods are hence advantageous. They offer better resolution based on genetic variability and enable the detection of recent transmission for outbreak control purposes (28). This relationship between genomic and phenotypic distance can be species specific, as differences in the level of concordance between WGS and FTIR have been observed between gram-negative organisms and *S. aureus* in outbreak analyses (9, 29). On the other hand, a change of a single SNP can result in a significant shift in the FTIR spectrum, resulting in the possible emergence of isogenic spectral variants (30).

However, FTIR did not result in any false-negative partitions, meaning that it did not align MRSA isolates from a single outbreak to separate clusters. This observation should be interpreted with caution, as the number of isolates in outbreak analysis was low, and metrics such as adjusted R and or adjusted Wallace coefficients could not be reliably applied. Nonetheless, this finding supports existing evidence that FTIR possesses adequate negative predictive power for *S. aureus*, which supports its proposed role as an inexpensive and rapid tool in preliminary detection of outbreaks, especially when a need for ruling out unrelated isolates arises (9, 16, 31). The possibility to reduce the number of isolates for WGS analysis could save resources during large or persistent outbreaks in resource-limited settings, or where a selective sequencing strategy has been applied, as is the case in Finland to date. However, based on the observed clustering pattern and the lack of standardized, stand-alone interpretation of the typing result for *S. aureus*, we believe that a preliminary FTIR clustering result should currently be confirmed with an alternative, preferably genotypic method.

A challenge of the method we encountered for *S. aureus* was the variation between technical but mostly biological replicates, resulting in six non-typeable isolates in the FTIR analysis (Table 1). This is a known challenge associated with the method and mitigated by careful standardization of the laboratory protocol (32). Additionally, the effect of the culture medium on the results can be significant, and tryptic soy agar has been a commonly used culture medium, with some evidence suggesting better performance than MH for *S. aureus* (17, 27). These variations can also arise between users, which can be reduced with further experience, since the acquisition range used in our study (1,300–800 cm^−1^) is believed to be less affected by culture conditions (12). Another challenge associated with the current FTIR methodology is related to scaling: as isolate count is increased above approximately 20 isolates, a supervised analysis strategy would be required to draw clinically meaningful inference.

Despite the limitations we faced, FTIR presented several advantages. The method was quickly established and compared to *spa* typing and especially WGS; FTIR was superior in terms of turnaround time and analysis cost per specimen.

In conclusion, while FTIR cannot replace high-resolution molecular methods of *S. aureus* strain typing, it continues to show promise as a rapid and cost-effective preliminary typing method for suspected MRSA outbreaks. Further research involving larger data sets, development of *S. aureus* analysis pipeline in the IR Biotyper, and further prospective evaluation are still needed to fully understand the utility of FTIR for the analysis of *S. aureus*.

## Data Availability

Raw sequence reads generated in this study have been deposited in the NCBI Sequence Read Archive under BioProject accession number PRJNA1372986. The isolate metadata and spectral data generated in this study have been deposited in Zenodo under DOI: 10.5281/zenodo.17802985.

## SUPPLEMENTAL MATERIAL

Supplemental figures spectrum.03245-25-s0001.pdf Fig. S1 to S16.

Supplemental tables spectrum.03245-25-s0002.xlsx Tables S1 and S2.

ASM does not own the copyrights to Supplemental Material that may be linked to, or accessed through, an article. The authors have granted ASM a non-exclusive, world-wide license to publish the Supplemental Material files. Please contact the corresponding author directly for reuse.
