# Supplementary material for: Evaluating the performance of Fourier transform infrared spectroscopy for typing and outbreak investigation of methicillin-resistant and -susceptible Staphylococcus aureus
Source: Microbiol Spectr. 2026 Apr 14;14(5):e03245-25. doi: 10.1128/spectrum.03245-25 (PMC13141934; doi:10.1128/spectrum.03245-25)
Supplement: Supplemental figures — Fig. S1 to S16. [file spectrum.03245-25-s0001.pdf]

Supplementary figures S1-S15 Results of the principal component analysis (PCA) model, *S. aureus* isolates by *spa* type (**Figures S1–S14**) and *S. aureus* isolates, singleton *spa* types (**Figure S15**). Each dot representing an absorption spectrum in the wave number region 1300–800 cm^{-1}. Six spectra, including two biological and three technical replicates of each isolate included, displayed in scatter plots using PC1 and PC2. **Figure S16** illustrates the result of the linear discriminant analysis of the outbreak isolates as a dendrogram.

Content:

Figure S1 t359 isolates. 2

Figure S2 t127 isolates. 2

Figure S3 t008 isolates. 3

Figure S4 t304 isolates. 3

Figure S5 t386 isolates. 4

Figure S6 t172 isolates. 4

Figure S7 t692 isolates. 5

Figure S8 t267 isolates. 5

Figure S9 t002 isolates. 6

Figure S10 t355 isolates.......... 6

Figure S11 t015 isolates.......... 7

Figure S12 t084 isolates.......... 7

Figure S13 *spa* types with 3 isolates each. 8

Figure S14 *spa* types with 2 isolates each. 8

Figure S15 Singleton *spa* types.......... 9

Figure S16 Linear discriminant analysis of the suspected outbreak isolates. 10

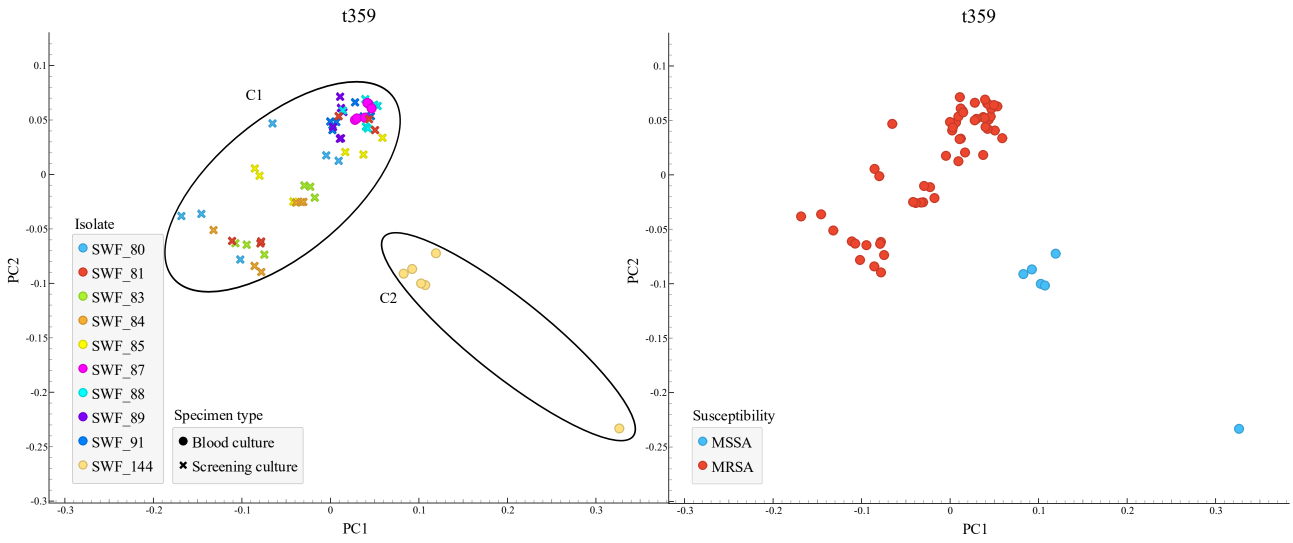

Figure S1 t359 isolates. Two-dimensional scatter plots of the PCA model annotated by isolate, specimen type and methicillin susceptibility, *spa* t359 isolates (n=10). Possible subtypes outlined (C1, C2), including nine (C1) and one (C2) isolates respectively. Spectra of a possible outlier, MSSA isolate SWF_144, separated from t359 MRSA isolates.

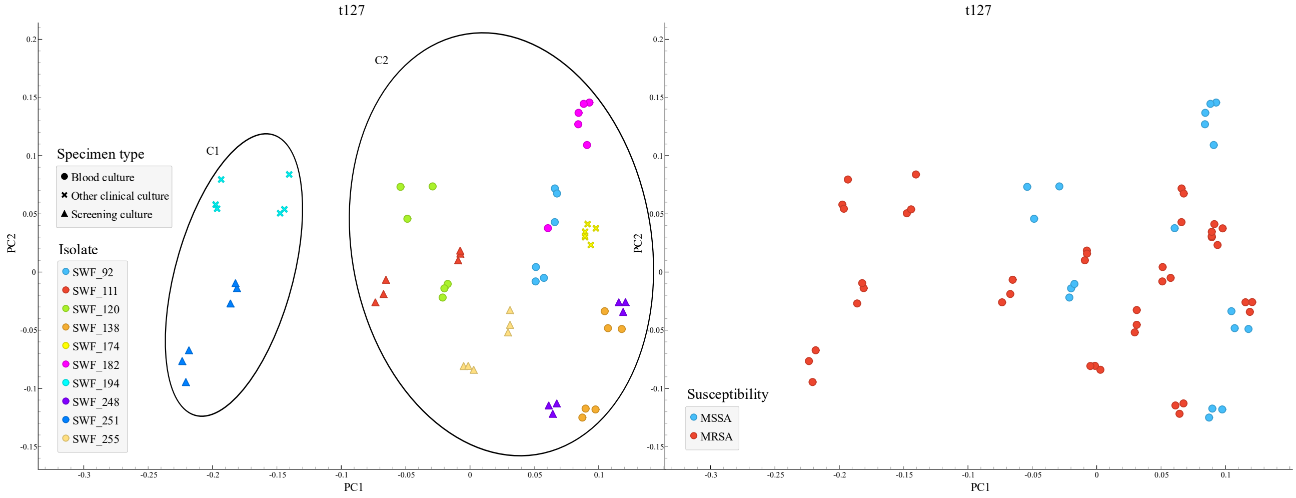

Figure S2 t127 isolates. Two-dimensional scatter plots of the PCA model annotated by isolate, specimen type and methicillin susceptibility, *spa* t127 isolates (n=10). Possible subtypes outlined (C1, C2), including two (C1) and eight (C2) isolates respectively. Overlap of spectra from MRSA and MSSA isolates observed in C2.

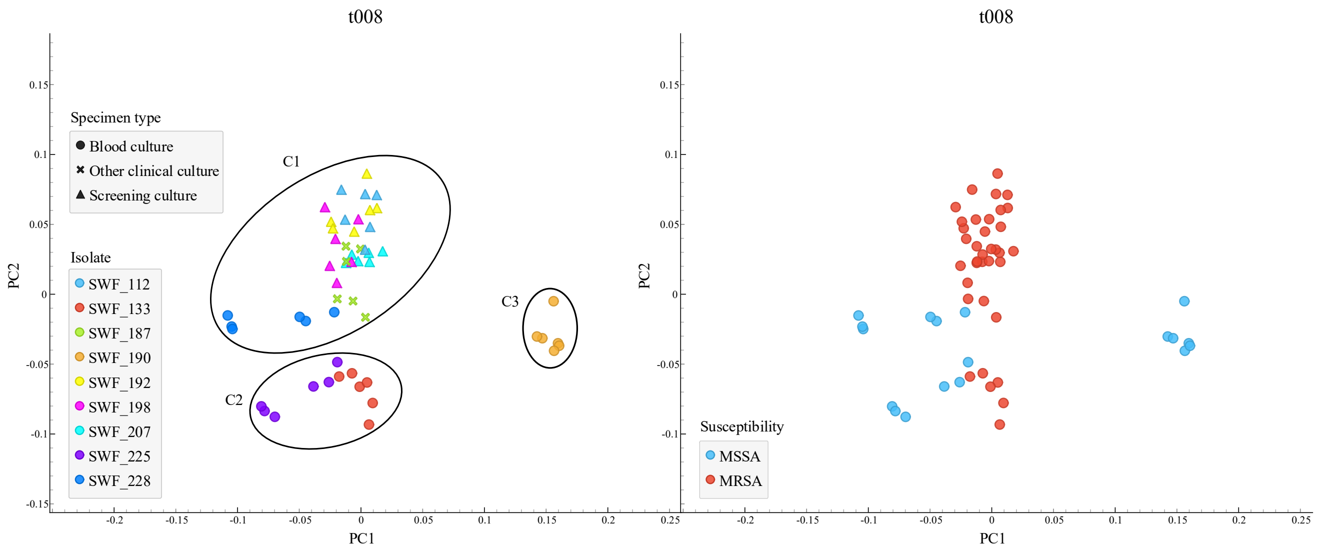

Figure S3 t008 isolates. Two-dimensional scatter plots of the PCA model annotated by isolate, specimen type and methicillin susceptibility, *spa* t008 isolates (n=9). Possible subtypes outlined (C1–C3), including six (C1), two (C2) and one isolate (C3), respectively. Overlap of spectra from MRSA and MSSA isolates observed in C1 and C2.

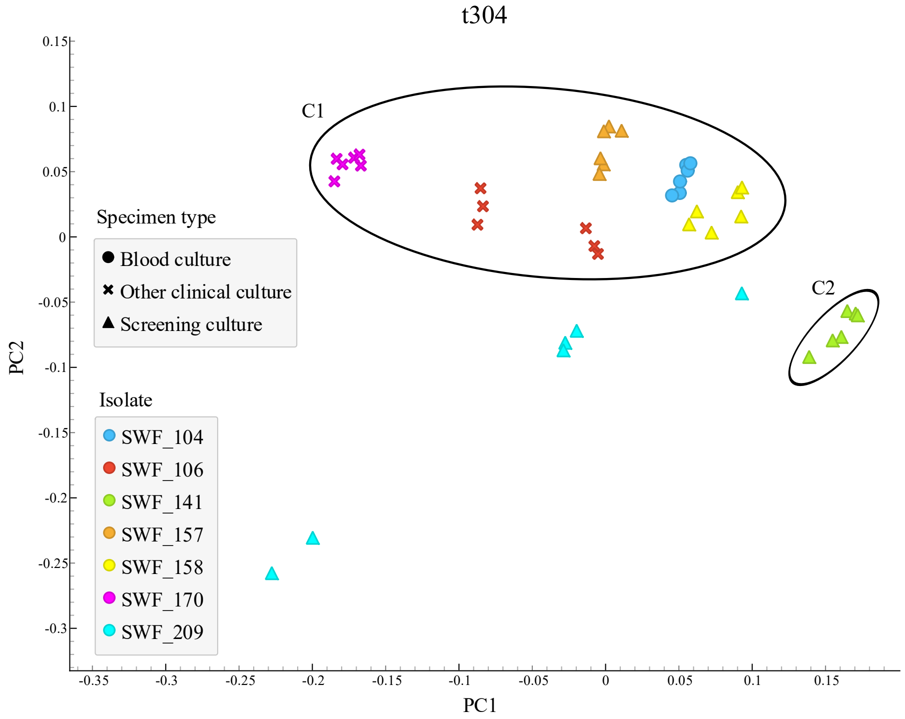

Figure S4 t304 isolates. Two-dimensional scatter plot of the PCA model annotated by isolate and specimen type, *spa* t304 isolates (n=7). All t304 isolates were MRSA isolates. Possible subtypes outlined (C1, C2), including five (C1) and one (C2) isolate, respectively. Isolate SWF_209 was considered nontypeable due to high technical variation.

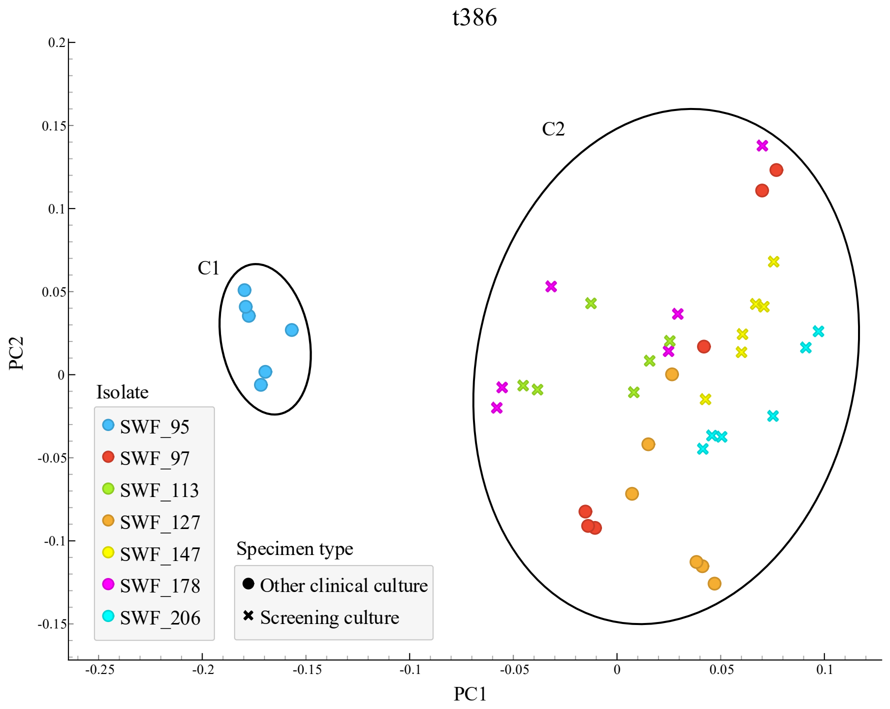

Figure S5 t386 isolates. Two-dimensional scatter plot of the PCA model annotated by isolate and specimen type, *spa* t386 isolates (n=7). All t386 isolates were MRSA isolates. Possible subtypes outlined (C1, C2), including one (C1) and six (C2) isolates, respectively.

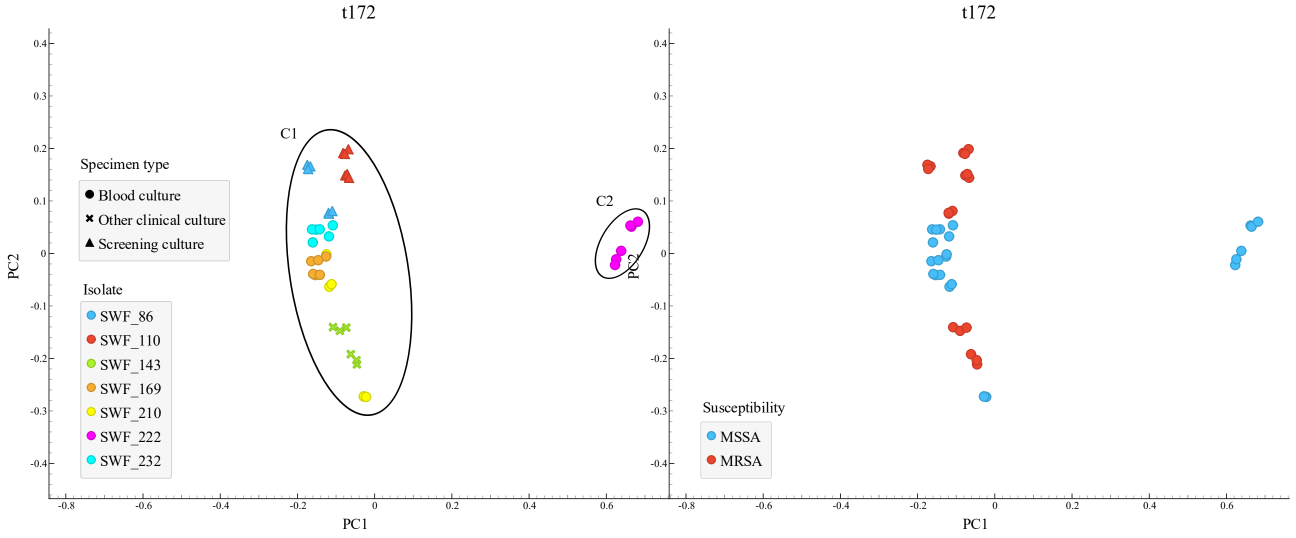

Figure S6 t172 isolates. Two-dimensional scatter plots of the PCA model annotated by isolate, specimen type and methicillin susceptibility, *spa* t172 isolates (n=7). Possible subtypes outlined (C1, C2), including six (C1) and one (C2) isolate, respectively. Overlap of spectra from MRSA and MSSA isolates observed in C1. Isolate SWF_86 was associated with suspected outbreak A (see **Figure 3A/B**).

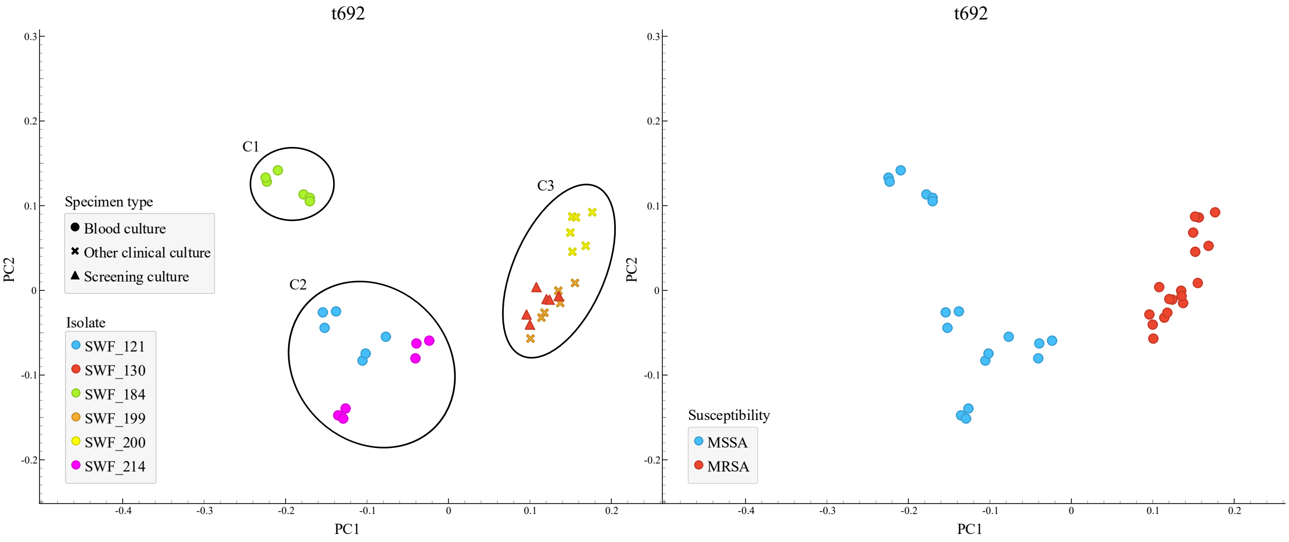

Figure S7 t692 isolates. Two-dimensional scatter plots of the PCA model annotated by isolate, specimen type and methicillin susceptibility, *spa* t692 isolates (n=6). Three possible subtypes outlined (C1–C3), where two MRSA isolates (C3) are separated from the possible MSSA subtypes (C1, C2) including one and two isolates, respectively.

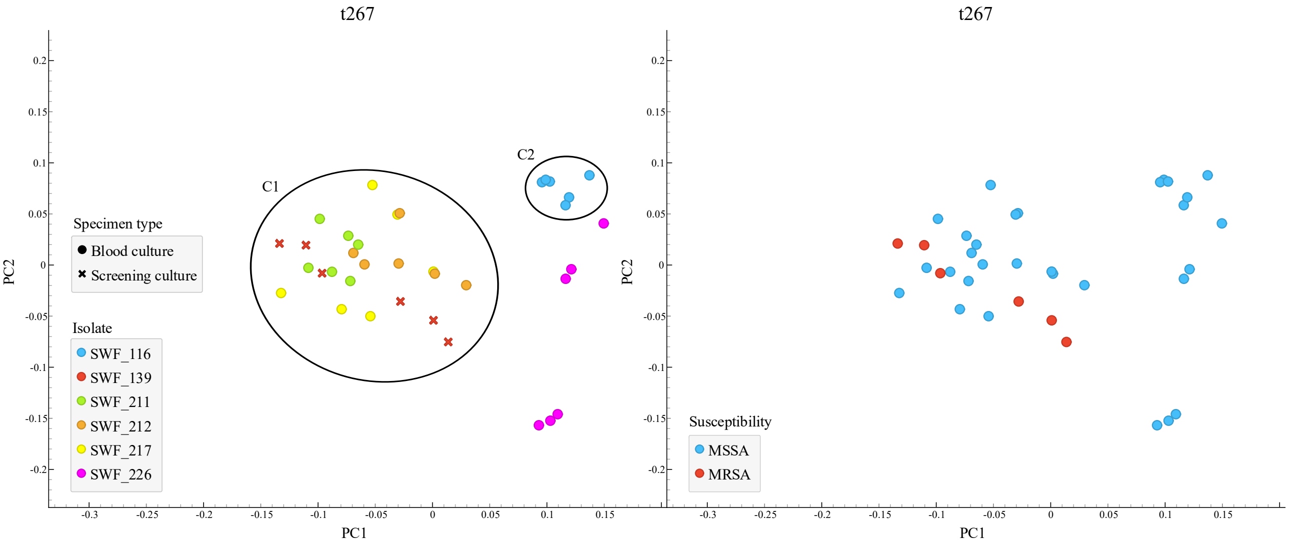

Figure S8 t267 isolates. Two-dimensional scatter plots of the PCA model annotated by isolate, specimen type and methicillin susceptibility, *spa* t267 isolates (n=6). Possible subtypes outlined (C1, C2), including four (C1) and one (C2) isolate, respectively. Overlap of spectra from MRSA and MSSA isolates observed in C1. SWF_226 was considered nontypeable due to high technical variation.

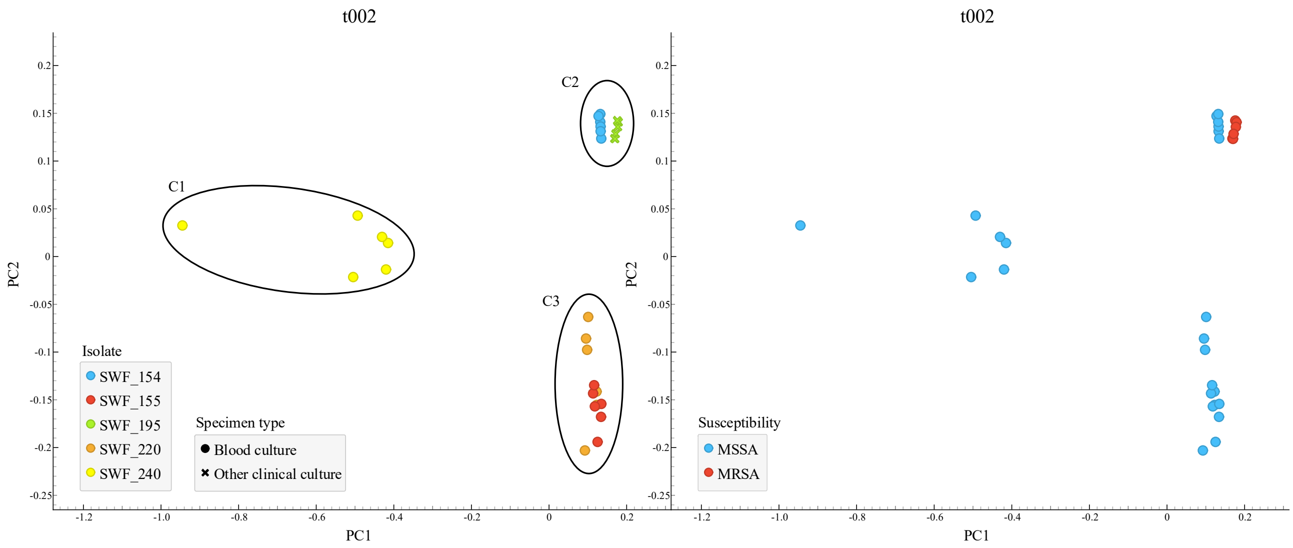

Figure S9 t002 isolates. Two-dimensional scatter plots of the PCA model annotated by isolate, specimen type and methicillin susceptibility, *spa* t002 isolates (n=5). Three possible subtypes outlined, one (C2) including very similar MRSA and MSSA isolates: SWF_154 and SWF_195, while other possible subtypes (C1, C3) consist of one or two MSSA isolates.

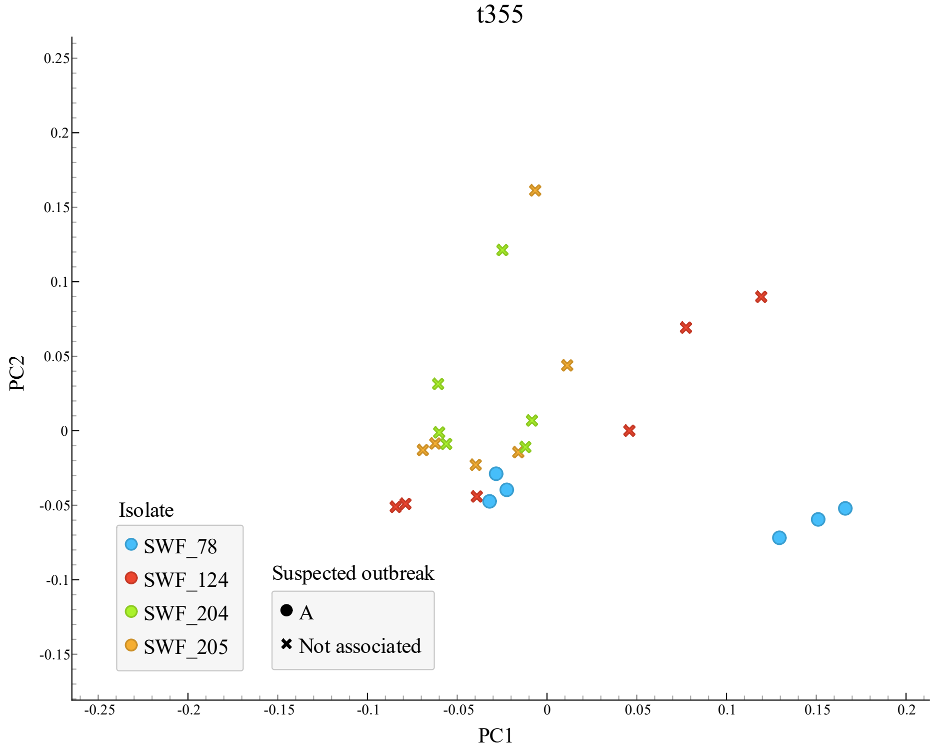

Figure S10 t355 isolates. Two-dimensional scatter plot of the PCA model annotated by isolate and suspected outbreak association, *spa* t355 isolates (n=4). All t355 isolates were MRSA isolates from screening specimens. Isolate SWF_78 was associated with suspected outbreak A (see **Figure S1**).

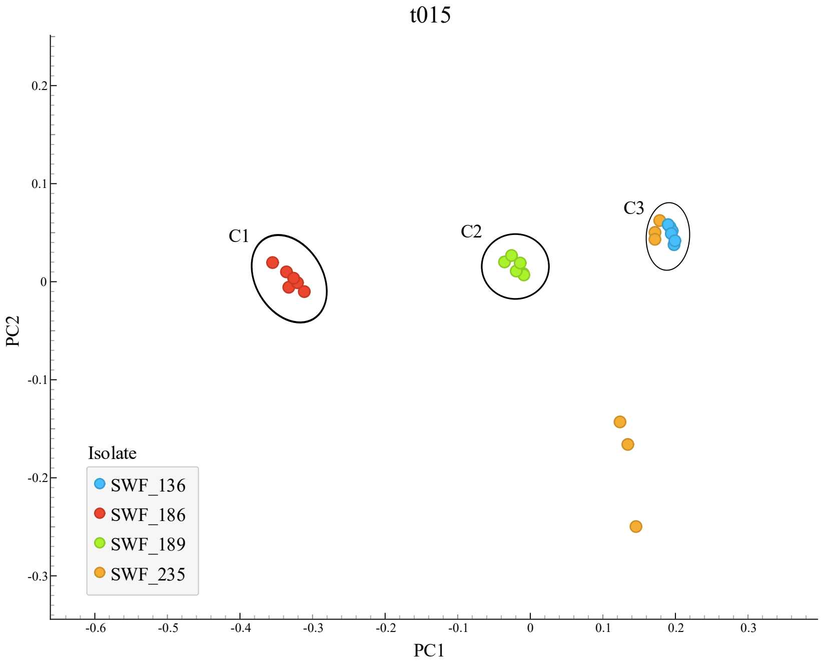

Figure S11 t015 isolates. Two-dimensional scatter plot of the PCA model annotated by isolate, *spa* t015 isolates (n=4). All t015 isolates were blood culture MSSA isolates. Three possible subtypes outlined (C1–C3), each containing the spectra of a single isolate. SWF_235 was considered nontypeable due to high technical variation.

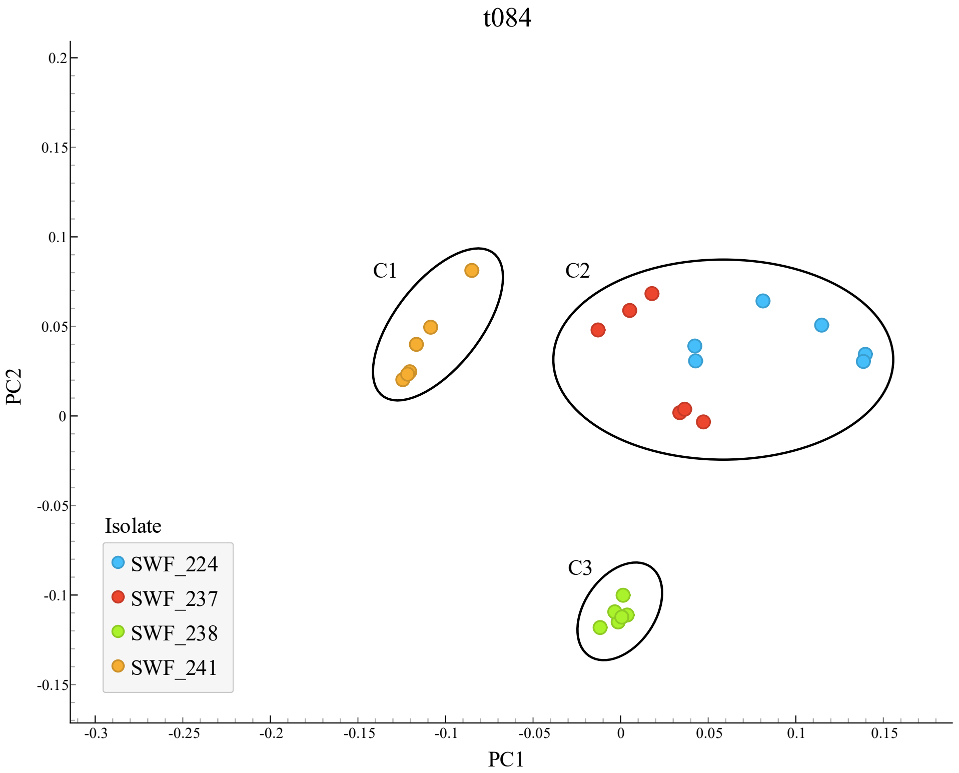

Figure S12 t084 isolates. Two-dimensional scatter plot of the PCA model annotated by isolate, *spa* t084 isolates (n=4). All t084 isolates were blood culture MSSA isolates. Three possible subtypes outlined, each containing spectra of a single (C1, C3) or two (C2) isolates.

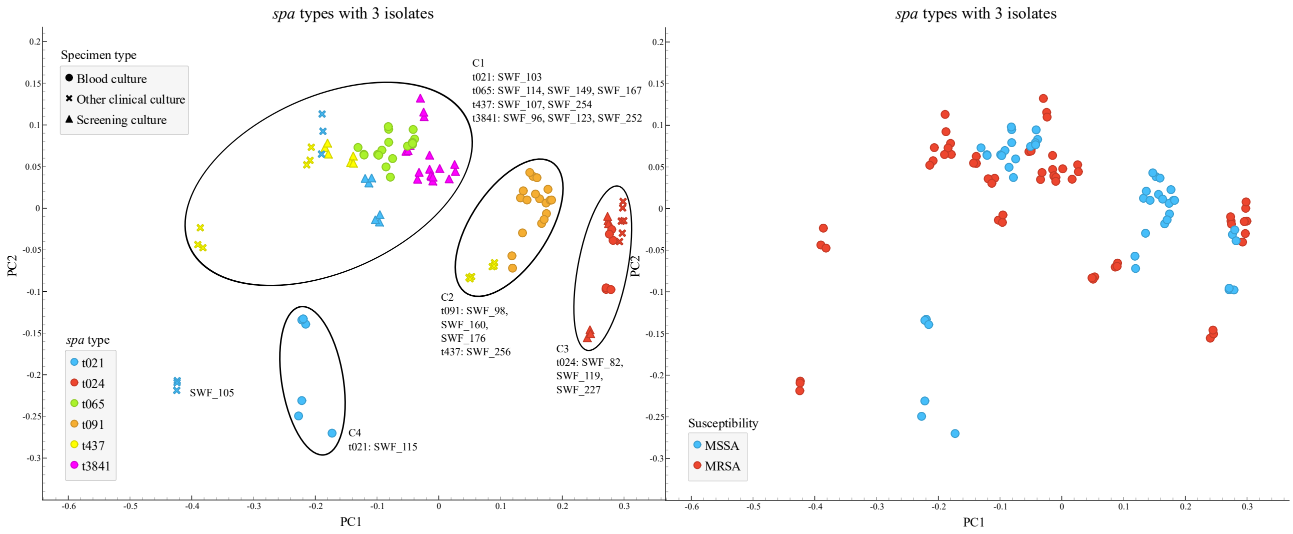

Figure S13 *spa* types with 3 isolates each. Two-dimensional scatter plot of the PCA model annotated by *spa* type, specimen type and methicillin susceptibility, isolates of 6 *spa* types with 3 isolates each (n=18). Four possible subtypes outlined (C1–C4). Isolates of *spa* t021 and t437 separated to different subtypes. Isolates sharing either *spa* types t024, t065, t091 and t3841 were not separated from other isolates of the same *spa* type. MRSA isolates SWF_107 and SWF_105 considered nontypeable due to high technical variability.

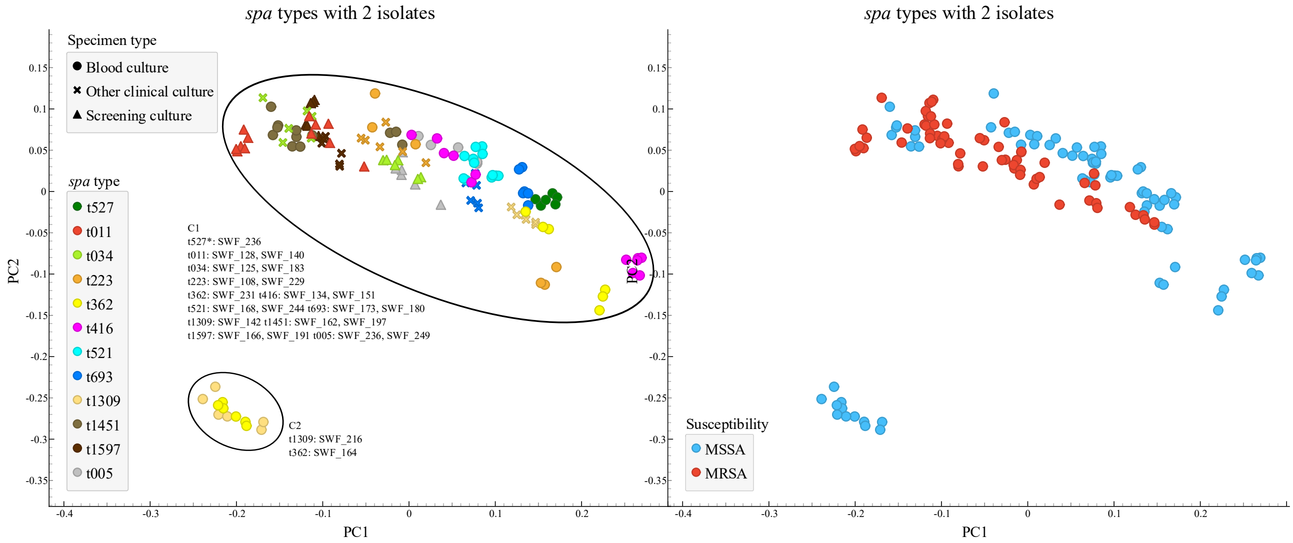

Figure S14 *spa* types with 2 isolates each. Two-dimensional scatter plot of the PCA model annotated by *spa* type, specimen type and methicillin susceptibility, isolates of 12 *spa* types with 2 isolates each (n=23) and a singleton t527 isolate SWF_236 (*the other t527 isolate SWF_245 not shown). Two possible subtypes outlined (C1, C2). Isolates of t1309 and t362 separated to different subtypes, where t1309 included an MRSA (wound swab) and an MSSA (blood culture) isolate. Otherwise, overlap of spectra from MRSA and MSSA isolates could be observed. Isolates sharing *spa* types t011, t034, t223, t416, t521, t693, t1451, t1597 and t005 were not separated from other isolates of the same *spa* type. SWF_245 was considered nontypeable due to high technical variation.

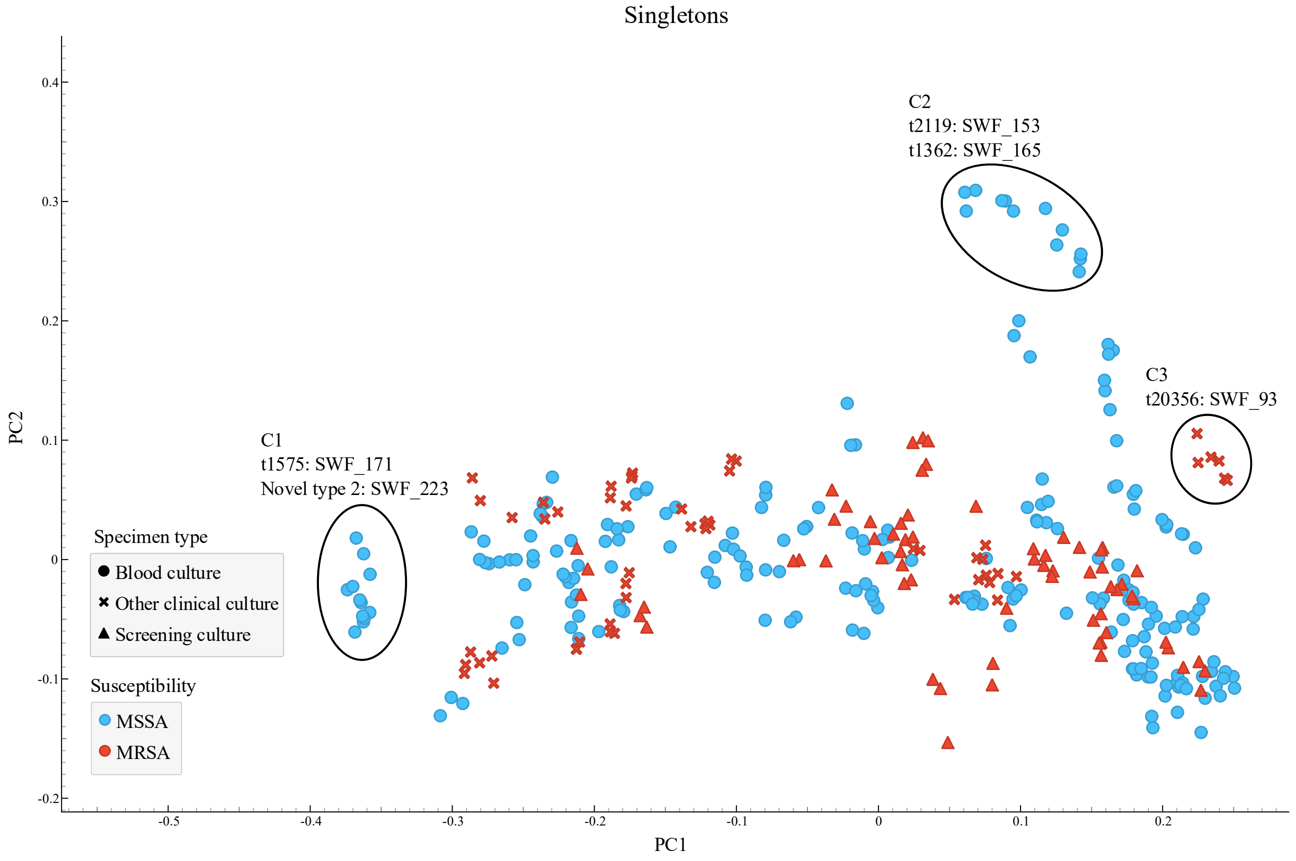

Figure S15 Singleton *spa* types. Two-dimensional scatter plot of the PCA model annotated by specimen type and methicillin susceptibility, isolates of 56 singleton *spa* types. Three possible subtypes outlined (C1–C3). Two subtypes (C1, C2) include only MSSA isolates while one subtype (C3) includes one MRSA isolate. Overall, overlap of spectra from MRSA and MSSA isolates could be observed.

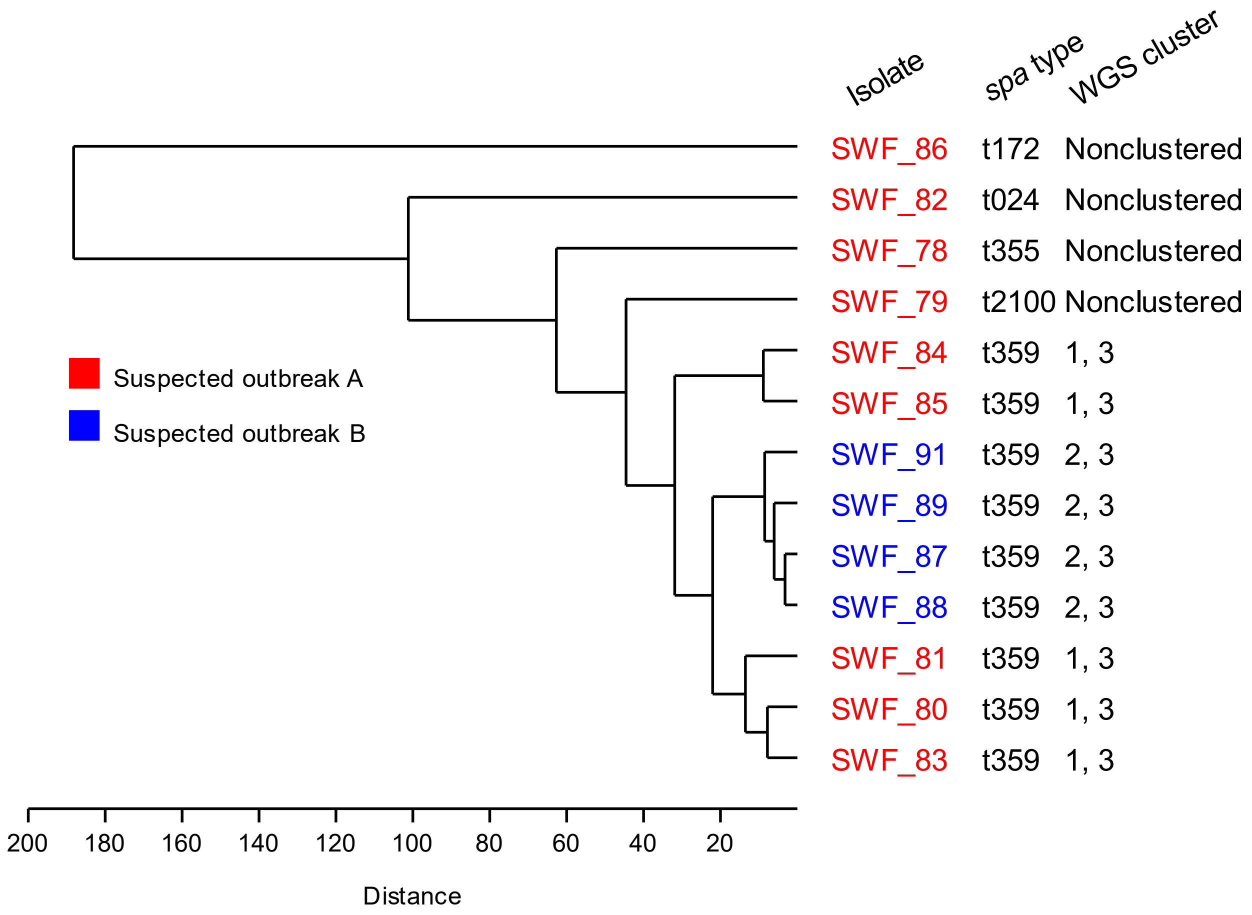

Figure S16 Linear discriminant analysis of the suspected outbreak isolates. A partition corresponding to the WGS analysis was observed, as nonclustered isolates (WGS cluster) were separated to different phyla from the t359 outbreak isolates. Analysis done with the PAST v.5.2 software.
